# Effectiveness of Multicentric Therapy Post Posterior Cervical Decompression and Fusion for Cervical Disc Disease: A Case Report

**DOI:** 10.7759/cureus.30544

**Published:** 2022-10-21

**Authors:** Dhanashree V Ghive, Shivani R Uttamchandani, Pratik Phansopkar

**Affiliations:** 1 Musculoskeletal Physiotherapy, Ravi Nair Physiotherapy College, Datta Meghe Institute of Medical Sciences, Wardha, IND

**Keywords:** spinal surgery, physiotherapy rehabilitation, posterior cervical decompression and fusion, cervical radiculopathy, cervical disc disease

## Abstract

In clinical practice, cervical disc abnormalities such as herniated nucleus pulposus (HNP), degenerative disc disease (DDD), and internal disc disturbance are seen. DDD includes degeneration causing annular tears, a decrease in disc height, and nuclear degradation. Cervical stenosis with myelopathy can be caused by anything that narrows the spinal canal and compresses the spinal cord, such as bone spurs, herniated discs, or bulging ligaments.

Posterior cervical decompression and fusion (PCDF) is a standard surgical treatment for treating numerous cervical spine diseases. It has been suggested that more intensive and structured physiotherapy is required to improve clinical outcomes with regard to long-term activity restrictions and participation restrictions and deficits in patients’ physical abilities relating to their neck post-surgery. In this case study a patient presented with complaints of upper back pain with a tingling sensation in the bilateral upper limbs for one year and was diagnosed with cervical disc disease with degenerative changes in the cervical spine along with disc bulges at C3-C4, C4-C5, C5-C6 levels causing severe spinal canal stenosis at C3-C4, C4-C5 disc levels and radiculopathy and then underwent a spinal fusion with posterior decompression surgery at the C4-C5-C6 level.

After surgery, the patient was started with physiotherapy rehabilitation which was planned for 12 weeks. Outcome measures that were included to record the recovery of the patient are Neck Disability Index (NDI) and the Numeric Pain Rating Scale (NPRS). Physiotherapy rehabilitation following posterior cervical spine decompression and fusion surgery for cervical disc disease has been proven to be beneficial in restoring the Range of Motion (ROM), and muscular strength of the bilateral upper limbs, reducing pain, and helping the patient get back to performing activities of daily living (ADLs) independently.

## Introduction

In clinical practice, cervical disc abnormalities such as herniated nucleus pulposus (HNP), degenerative disc disease (DDD), and internal disc disturbance are seen. DDD includes degeneration causing annular tears, a decrease in disc height, and nuclear degradation. Cervical spine disease is frequent in adults, with a frequency of approximately 95% by the age of 65 years. Despite the fact that different biological variables have been linked to disc disease, improper spinal motion, and, as a result, abnormal loading has been thought to be the cause of spinal pathology. Spinal disorders can cause various disc diseases like instability, neurological deficit, spinal stenosis, and facet dysfunction. These clinical symptoms might cause individuals to lose neck mobility and become disabled [1]. The nucleus pulposus of the intervertebral disc shifts at the cervical level as a result of cervical disc herniation, which may directly compress the spinal cord or entrap nerve roots [2]. Depending on the extent of disc protrusion, this disc herniation has a variety of consequences. Cervical disc herniation often develops when the forces of flexion, extension, rotation, and combination exceed the annulus fibrosis strength. Some of the common symptoms include ipsilateral pain in the neck or pain that radiates down the ipsilateral arm to the fingers [1]. Cervical stenosis with myelopathy can be caused by anything that narrows the spinal canal and compresses the spinal cord, such as bone spurs, herniated discs, or bulging ligaments [3]. The most common early signs of spinal canal stenosis are abnormal sensations in the hands, altered gait, and impairments in the fine motor abilities of the hands. Difficulty in writing is most common at advanced stages. Eventually, the hands are completely incapable of holding anything [4]. Posterior cervical decompression and fusion (PCDF) is a standard surgical procedure for treating numerous cervical spine diseases The procedure of posterior cervical decompression and fusion enables the decompression of several cervical spine segments in patients with multi-level stenosis. It involves inserting screws into the lateral masses, which are connected by a rod to offer quick stability and fusion [5].

Based on the chosen outcome measure and the period of follow-up, there have been reports of average to excellent outcomes in terms of arm pain and neurological symptoms following surgery. The effects on neck function are less clear, and it has been suggested that more intensive and structured physiotherapy is required to improve clinical outcomes with regard to long-term activity restrictions and participation restrictions and deficits in patients' physical abilities relating to their neck post-surgery. Patients may be prescribed structured physical therapy before and after surgery that combines behavioral therapy with exercises designed specifically for the neck [6].

## Case presentation

Patient information

A 33-year-old male patient visited the hospital with complaints of upper back pain with a tingling sensation in the bilateral upper limb for one year and weakness in the same for four months. The pain was insidious in onset and gradually progressive and radiating to his bilateral upper limbs. The pain has started aggravating over the past three to four months. He had no history of trauma to the back, fall, or recent heavy weight lifting. He had a history of trauma to the right wrist one year back. The patient was then evaluated and underwent investigations like X-ray, Magnetic Resonance Imaging (MRI), and Computed Tomography scan (CT scan) of the cervical spine which was suggestive of straightening of the cervical spine, degenerative changes in the cervical spine with disc bulges at C3-C4, C4-C5, C5-C6 disc levels causing severe spinal canal stenosis at C3-C4, C4-C5 disc levels and radiculopathy and ossified posterior longitudinal ligament. The patient was then planned for spinal fusion with posterior decompression surgery at the C4-C5-C6 level. After successful surgery patient was shifted to the ward and was referred to the physiotherapy department on post-operative (POD) day four for further management.

Clinical findings

A thorough clinical examination was done, the patient was examined in a supine position and he was cooperative, conscious, and oriented to time, place, and person. He was hemodynamically stable. Pain history was taken, and he rated the pain as 8/10 on activity and 6/10 on rest on the numeric pain rating scale (NPRS) over the upper back and during cervical joint movements. He also complained of bilateral (B/L) upper limb weakness leading to difficulty in performing activities of daily living and decrease ROM of the cervical joint. He felt pain at the suture site when he moved his neck, but it did not radiate to his arm. The initial active cervical range of motion was evaluated by gross examination and classified as zero, mild, moderate, or significant. On palpation, paraspinal muscle spasms and soft tissue tenderness were present. The range of motion of bilateral upper limbs was assessed and it was found to be reduced (for ROM refer to Table 1) and B/L lower limbs were found within acceptable ranges. To determine whether any peripheral weakness resulting from a cervical nerve impingement existed, myotomes were tested. The patient was negative for any nerve root involvement. Manual Muscle Testing (MMT) was also performed to document the strength of the bilateral upper limb and it was found to be reduced (for MMT refer to Table 2).

**Table 1:** Range of motion values

Joint	Pre Treatment			Post Treatment			
Cervical Flexion	30°			70°			
Cervical Extension	25°			55°			
Cervical lateral flexion	10°			30°			
	right	left		right	left		
Shoulder flexion	80°	75°		140°		145°	
Shoulder extension	10°	12°		40°		35°	
Shoulder abduction	70°	65°		150°		147°	
Shoulder adduction	10°		12°	35°			40°
Elbow flexion	70°		75°	120°			115°
Wrist Flexion	20°		25°	60°			65°
Wrist Extension	25°		30°	65°			68°

**Table 2:** MMT grades MMT: Manual Muscle Testing

Muscles	Pre Treatment	Post Treatment
Shoulder Flexors	1/5	4/5
Shoulder Extensors	1/5	4/5
Shoulder Abductors	1/5	4/5
Shoulder Adductors	2/5	4/5
Elbow Flexors	2/5	4/5
Elbow Extensors	1/5	4/5
Wrist Flexors	1/5	4/5
Wrist Extensors	1/5	4/5

Timeline

The patient was admitted on 23/02/2022 and the surgery was done on 04/03/2022. Physiotherapy rehabilitation was started on 08/03/2022.

Diagnostic assessment

**Figure 1:**
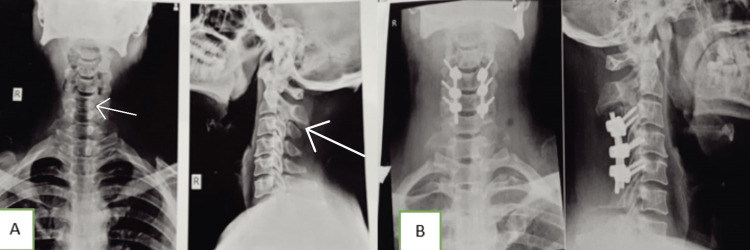
Pre- and post-operative radiogram images (A) Pre-operative – showing PA (left-hand side) and lateral view (right-hand side), (B) Post-operative – showing PA (left-hand side) and lateral view (right-hand side). Postero-anterior (PA), Antero-posterior (AP)

Radiogram of the cervical spine (antero-posterior {AP} and lateral): There is evidence of radio-opaque mass causing possible destruction of the C1 vertebra (Figure 1).

MRI Cervical Spine

**Figure 2:**
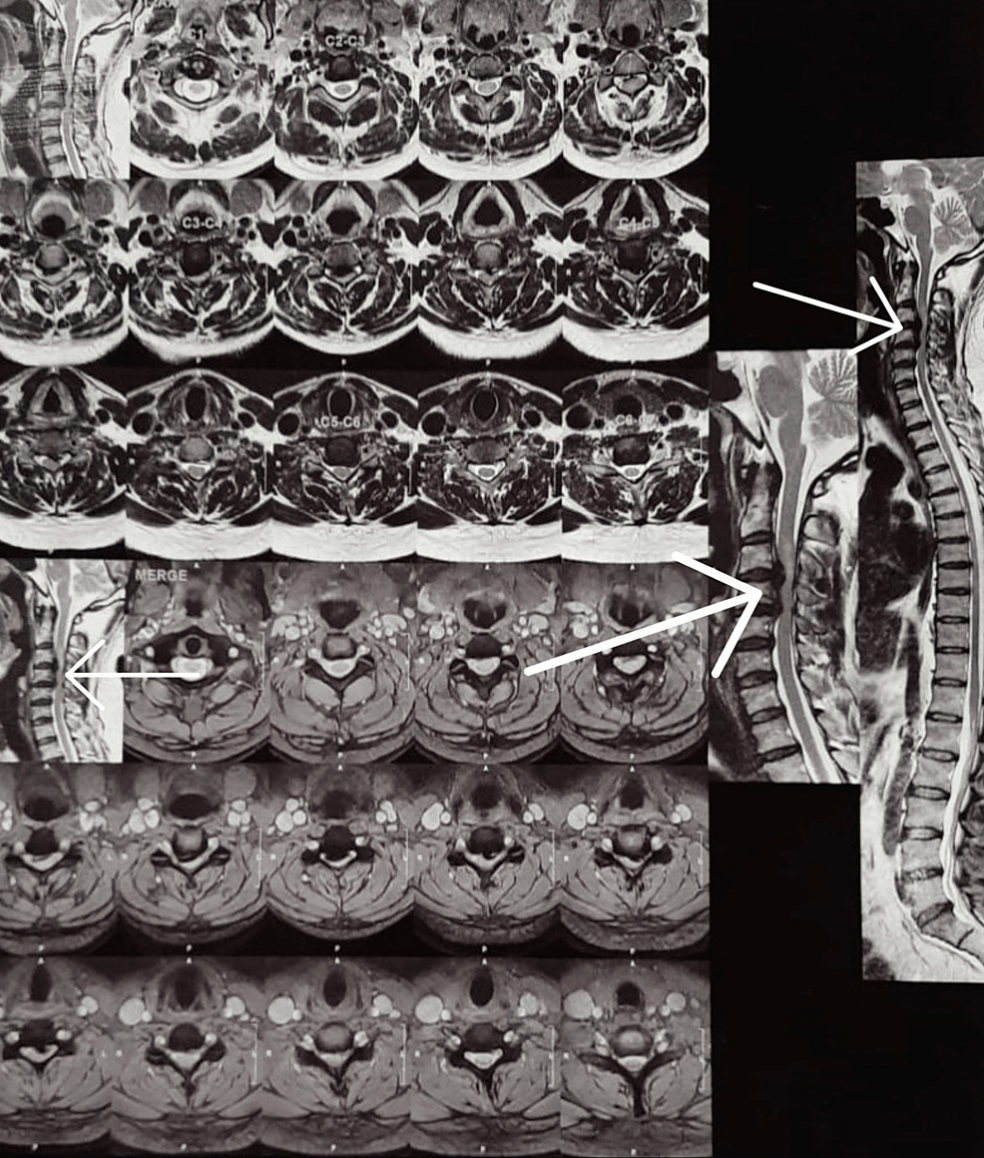
MRI image showing disc bulge Arrows showing areas of disc bulge

**Figure 3:**
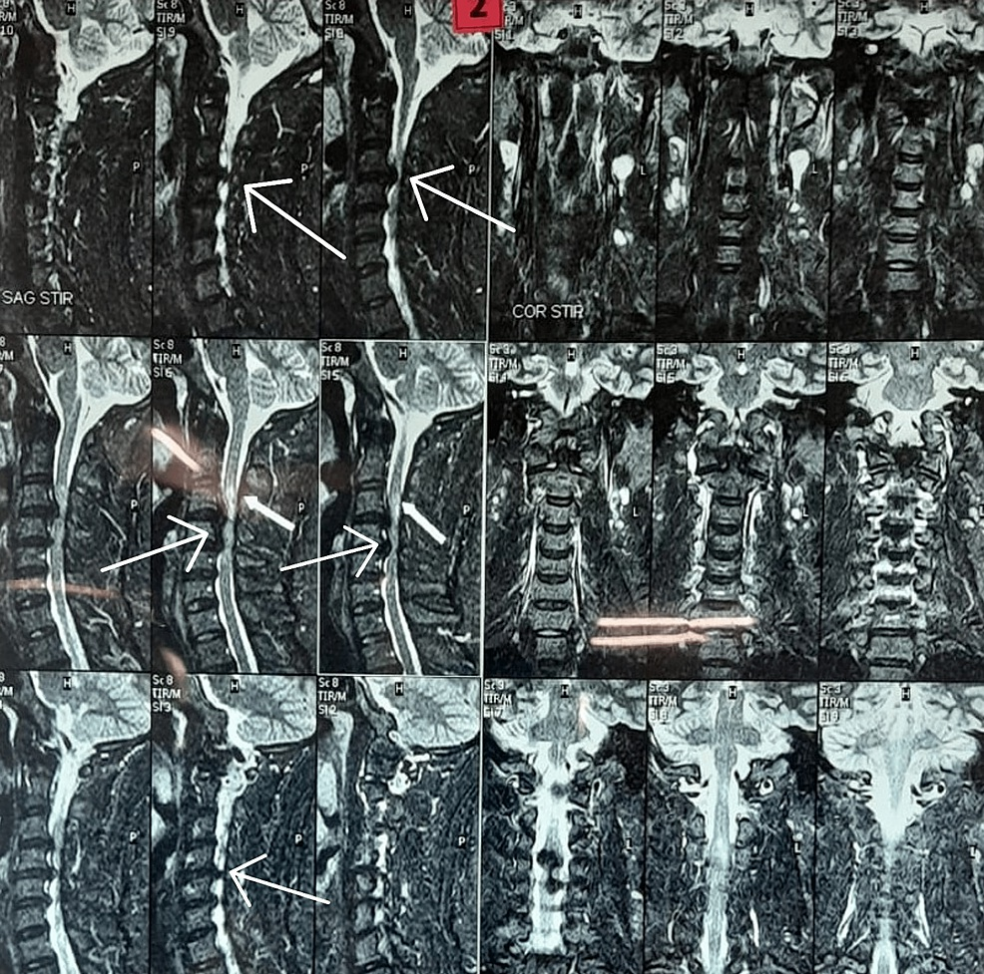
MRI images showing disc bulge Arrows are showing the disc bulges

Altered curvature of the cervical spine, cervical spine degenerative changes with disc bulges at C3-C4, C4-C5, and C5-C6 disc levels causing severe spinal canal stenosis at C3-C4, C4-C5 disc levels, and radiculopathy (Figures 2, 3).

Canal Diameter

Pre-operatively at the C3-C4 disc level, the canal diameter was 4.7 mm, as well as, at the C4-C5 disc level, the canal diameter was 5 mm.

Therapeutic intervention

Following the surgical procedure, the patient was started with physiotherapy rehabilitation which was planned for 12 weeks. The patient was explained about the rehabilitation program and its benefits. The goal of the treatment was to improve function, and the performance of basic activities, prevent stiffness, re-educate movement patterns and give postural education, and also improve stabilization. The treatment plan was focused on improving upper extremity function mobility and strength, avoiding complications related to surgery, and making the patient independent to perform his activities of daily living. After the surgical procedure patient gained some upper limb control. Now the goal was to increase the upper limb strength to the fullest.

During the initial four weeks, firstly the patient was educated about the importance of exercise following surgery to avoid complications and to get back to activities of daily living as early as possible. The patient was given ergonomic advice while performing normal activities like avoiding bending activities, sitting using a chair with a proper backrest, avoiding activities that will put a strain on the cervical spine, and rolling in bed using the log rolling technique. To decrease pain and inflammation after surgery ice pack application for 2-6 minutes two times a day was advised. He was educated about bed mobility and transfers and spine stabilization while performing activities. Initially, the patient was allowed to sit with a lumbar roll in a chair or at the bedside with a cervical collar for not more than 30 minutes. He was taught breathing (diaphragmatic breathing) and relaxation exercises and was advised to perform them three to four times a day for 1-2 minutes to avoid postoperative pulmonary complications. To avoid the complications related to prolonged bed rest, the patient was taught isometric exercises of lower limbs like isometrics to quadriceps, hamstring, and gluteal muscles and ankle pumps to increase blood circulation twice a day with three sets of 10 repetitions. He was also encouraged to walk two times a day as per tolerance.

For the next four to eight weeks, the patient was advised to continue with breathing exercises and was taught how to maintain an upright posture. Exercises for active cervical range of motion were started while limiting end ranges to release constraints for cervical flexion, extension, lateral flexion, and rotation and so obtain gross cervical movement. Exercises were given for cervical stabilization with increasing limb loading. To increase the neuromuscular control of the deep cervical flexors, the patient was started with neck isometrics and scapular retraction exercises. Scapular movement re-education including shoulder shrugs, shoulder rolls, and scapular mobilization exercises was initiated with gentle isometrics to cervical extension, flexion, rotation, and side bends given in a sitting position. After that progression, upper thoracic mobilization activities such as cat-and-camel maneuvers, upper thoracic extension, upper thoracic rotation, arm clocks, and combined thoracic and cervical movement were performed. Longus Colli was subjected to neuromuscular re-education with pressure biofeedback, which involved arm and leg movements in various positions with a weight limit of 5 lbs, along with it abdominal exercises, and basic core strengthening of the lumbar spine was done. Cardiovascular training was also initiated with a treadmill and stationary bike as per tolerance.

After this for the next few weeks, treatment included strengthening the upper limb muscles, initially holding 0.5 kgs of weight, and performing horizontal abduction-adduction, flexion-extension, and elbow flexion-extension as per tolerance with one set of 10 repetitions. When the patient was successfully able to perform this without any increase in pain and symptoms he was advised to perform exercises by performing two sets and eventually increasing the weight.

Outcome measures 

**Figure 4:**
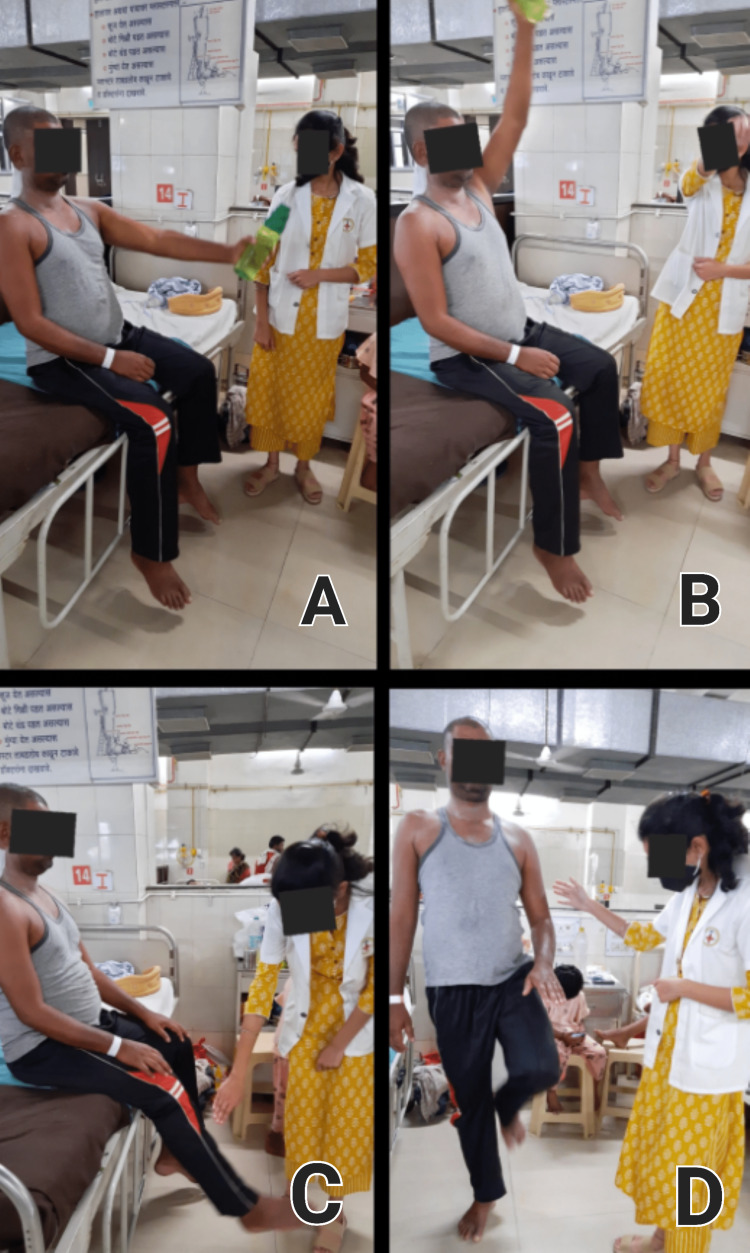
Images of the patient performing strengthening exercises A and B: Patient performing strengthening exercises (flexion-extension), C: Patient performing seated knee extension, and D: Patient performing spot marching.

Following this treatment, the patient began to gain upper limb strength and was able to perform the activities of daily living (ADLs) independently. The patient was also explained in detail about the home exercise program and its importance so that he will continue the exercise at home on his own and will gain full strength. Thus following this rehabilitation patient showed improvement in his range of motion, strength, stabilization, and performance in ADLs. Pre- and post-intervention outcome measures are mentioned in Table 3. Patient performing exercises such as strengthening exercises, lower limb exercises, and spot marching is shown all together in a collage in Figure 4.

**Table 3:** Outcome measures

Outcome Measures		Pre-test	Post-test
Neck Disability Index		52 %	14 %
Numeric Pain Rating Scale		8/10	1/10
Patient-specific functional scale	Picking up an object	3	10
	Grasping an object	3	9

## Discussion

Cervical disc herniation is a problem that rarely responds to a single course of therapy. It is a progressive disorder that typically manifests as pain and numbness in the neck and arm, as well as ipsilateral diverging discomfort that correlates to damaged cervical levels. Surgery has frequently been employed as a last option for treating cervical disease. In this case study patient presented with complaints of upper back pain with a tingling sensation in the bilateral upper limb for one year and was diagnosed with cervical disc disease with degenerative changes in the cervical spine with disc bulges at C3-C4, C4-C5, C5-C6 disc levels causing severe spinal canal stenosis at C3-C4, C4-C5 disc levels and radiculopathy and then underwent a spinal fusion with posterior decompression surgery at the C4-C5-C6 level. Therefore here, physiotherapy rehabilitation following posterior cervical spine decompression and fusion surgery for cervical disc disease has been shown to be important in restoring range of motion, and muscle strength, reducing pain, and helping the patient get back to performing ADLs independently.

Numerous studies have demonstrated that spinal stabilizer training is beneficial for treating both cervical and lumbar spine diseases, improving spinal mobility, and enhancing functional strength [7]. Patients with mechanical neck pain have responded favorably to interventions that have focused on restoring deep cervical muscle strength [7]. Similarly in our report, the cervical ROM of the patient was improved by performing a range of motion and stabilization exercises for the cervical joint. Adhesions and scar tissue began to relax without any pain at the suture site, and the soft tissue around his cervical and thoracic spine became more mobile. We believe this effect is brought on by the smaller stabilizing muscles because of their ability to directly stabilize the vertebral segments and reduce undue strain or stress on the intervertebral disc, which is important for stability in the cervical spine [7,8].

Patients with cervical disc disease and Cervical Radiculopathy may benefit from structured physical therapy that includes neck-specific exercises and a behavioral strategy both before surgery and during postoperative rehabilitation. The study by Wibault et al. revealed that patients with cervical radiculopathy can sustain neck-specific activities following surgery and make progress over time and compared to earlier trials, both groups at three months showed significant reductions in Neck Disability Index (NDI) and visual analog scale (VAS) scores for neck and arm pain [6].

## Conclusions

Physiotherapy rehabilitation following posterior cervical spine decompression and fusion surgery for cervical disc disease has been proven to be beneficial in restoring ROM, muscular strength of bilateral upper limbs, reducing pain, and helping the patient get back to performing ADLs independently. In our report, the cervical ROM of the patient was improved by performing a range of motion and stabilization exercises for the cervical joint. Adhesions and scar tissue began to relax without any pain at the suture site, and the soft tissue around his cervical and thoracic spine became more mobile. We believe this effect is brought on by the smaller stabilizing muscles because of their ability to directly stabilize the vertebral segments and reduce undue strain or stress on the intervertebral disc, which is important for stability in the cervical spine.
